# Edge enhancement improves disruptive camouflage by emphasising false edges and creating pictorial relief

**DOI:** 10.1038/srep38274

**Published:** 2016-12-06

**Authors:** John Egan, Rebecca J. Sharman, Kenneth C. Scott-Brown, Paul George Lovell

**Affiliations:** 1 Abertay University, Division of Psychology, School of Social and Health Sciences, 1, Bell St, Dundee, DD1 1HG, United Kingdom; 2 University of Stirling, Faculty of Natural Sciences, Department of Psychology, Stirling, FK9 4LA, United Kingdom

## Abstract

Disruptive colouration is a visual camouflage composed of false edges and boundaries. Many disruptively camouflaged animals feature enhanced edges; light patches are surrounded by a lighter outline and/or a dark patches are surrounded by a darker outline. This camouflage is particularly common in amphibians, reptiles and lepidopterans. We explored the role that this pattern has in creating effective camouflage. In a visual search task utilising an ultra-large display area mimicking search tasks that might be found in nature, edge enhanced disruptive camouflage increases crypsis, even on substrates that do not provide an obvious visual match. Specifically, edge enhanced camouflage is effective on backgrounds both with and without shadows; i.e. this is not solely due to background matching of the dark edge enhancement element with the shadows. Furthermore, when the dark component of the edge enhancement is omitted the camouflage still provided better crypsis than control patterns without edge enhancement. This kind of edge enhancement improved camouflage on all background types. Lastly, we show that edge enhancement can create a perception of multiple surfaces. We conclude that edge enhancement increases the effectiveness of disruptive camouflage through mechanisms that may include the improved disruption of the object outline by implying pictorial relief.

The camouflage of the copperhead snake (*Agkistrodon contortrix, see* Fig. 1 *top-left*) is composed of a pattern of irregular shapes in shades of brown; it provides an example of two ways to achieve crypsis. Firstly through background matching, where colour and pattern differences between the camouflaged object and its usual background of soil and leaves are minimised^1^,^2^. Secondly through disruptive colouration, where the pattern conceals the true shape of an object and encourages the perception of multiple segregated objects^3^,^4^. The contrast between pattern elements creates false edges^5^; and differential blending of the different pattern elements with the background, this encourages incorrect perceptual segregation of a pattern^6^.

Many animals with disruptive colouration feature accentuated borders between pattern elements^7^. For the spotted marsh frog (Limnodynastes tasmaniesis) the edges of the lighter pattern elements transition towards white, whereas the darker pattern elements transition towards black (see Fig. 1). The points where light and dark pattern elements meet are the regions of highest contrast in an edge enhanced camouflage pattern. For other species the edge enhancement may consist of only a dark (see Fig. 1. Jaguar) or light outline (Fig. 1, Lime Hawk Moth).

Hugh Cott^3^ and before him Abbot Thayer^8^ have both discussed the role of edge-enhancement in camouflage, albeit with a different terminology. We chose to present an image of a copperhead snake (Fig. 1, top-left) because this is the same species selected by Thayer (ref. 9 see Plate Xi, page 172) and Cott (3 Fig. 18, page 67) to illustrate how well-matched the sedentary snake’s colouration and pattern is to a background of dead leaves. Thayer describes how the snake colouration is formed from “background pictures” (9, page 175). Here he is describing the visual similarity between the snake patterning to a heap of leaves and their shading patterns. Elsewhere in his book, Thayer illustrates the similarity of various animal patterns to photographs of bark and fallen leaves (3 Fig. 119, page 159). Cott^3^ describes how an illusory impression of depth might be suggested by patterns of colouration that correspond to pictorial relief (especially Fig. 25 page 76).

In terms of the visual processes underlying object detection, the addition of enhanced edges to disruptive colouration may serve two roles. Firstly, V1 neurons, or the equivalent in other taxa, that detect edges are excited by abrupt changes in reflectance^10^. These signals are then used in later visual areas for image segmentation including parsing the visual input into distinct objects^11^. False edges that are emphasized by edge enhancement may encourage incorrect boundary segmentation, inhibiting detection and then accurate object recognition^7^. See Troscianko *et al*.^12^ for a discussion of the interplay between early visual processing and camouflage. Secondly, in addition to emphasising false edges, edge enhancement might also mislead depth perception by creating an impression of pictorial relief^3^,^13^. For example, velvety textured black spots on the West African Gaboon viper (*Bitus rhinoceros*; see ref. 14) give a strong impression of spatial depth by mimicking the appearance of shading in natural scenes^13^. Edge enhancement can create the perception that pattern elements occupy different relative depths. Therefore, edge enhancement may also play a role in disruption of depth perception. This could then disrupt those perceptual processes that use surface shading to inform estimates of object shape (see refs 15-17).

While the stimuli used in our study feature camouflaged snake-shaped targets, these were chosen in order to encourage participant engagement, in fact we are interested in a general principle of visual camouflage that may be applicable to many different cryptic species in a range of phyla. We do not attempt to model the camouflage of any specific species; instead we adopt a parsimonious model of edge enhancement with only two free parameters (see Fig. 2, bottom row). We then applied this edge enhancement to a simple model of disruptive camouflage (see Methods) and placed the camouflaged objects upon a cluttered background, which affords an approximate spatial and chromatic match to the target. A key feature of successful camouflage is that it is unlikely to attract close inspection from curious viewers. However, many experiments on camouflage actually employ relatively small displays, which result in close attention and perhaps foveated vision being built-in. Although it should be noted that there are instances where animals forage with a small viewing distance, i.e. birds looking at the bark of a tree. In the current study we employ a 6 metre wide display featuring a single target that is only 1/10^th^ of this along its longest axis. As a consequence, successfully camouflaged objects do not immediately attract close inspection, more closely replicating natural viewing. However, we have recently replicated the results of the current study on conventional, smaller-scale, display systems.

Initially we tested the effectiveness of symmetrical edge enhanced camouflage with a profile similar to that of the spotted marsh frog (see Fig. 1 top-right). In a search task, a target was set against a background of leaf objects (Fig. 2). In Experiment 1 we manipulated the degree of the target’s edge enhancement and the background appearance; including leaves that cast shadows or that did not cast shadows. This allows us to explore the effectiveness of edge-enhanced camouflage against backgrounds with and without shadows – exploring Thayer’s assertion that such patterns are an example of camouflage built from “background pictures”. In Experiment 2, we manipulated the structure of the edge enhancement by removing either the light or the dark edge, to further explore the contribution of edge enhancement towards background matching. We also remove the gradient of the edge enhancement, exploring whether edge enhancement with a square profile is as effective. Experiment 3 explored whether edge enhancement creates an impression of pictorial relief, and whether edge enhancement could allow for better background matching in terms of the amount of pictorial relief. This was tested by asking participants to judge the amount of pictorial depth in stimuli with varying degrees of edge enhancement.

## Experiment 1: Does edge enhancement increase the effectiveness of disruptive camouflage?

### Results and Discussion

Reaction times were log transformed, so that they had a normal distribution, and then all times for each participant were z-scored to reduce the impact of inter-participant variability. There was a strong positive correlation between reaction times and errors (see Supplemental Materials for details) suggesting that there was no speed-accuracy trade-off between conditions. On individual error-trials it is impossible to know whether participants are merely pressing the incorrect button after correctly identifying the target or if they have given up after a long search, so these trials were excluded from the analysis. For each participant the mean reaction time was calculated for each condition. In order to look at the effects of edge enhancement the means for the control (no-edge enhancement) condition were subtracted from the means for each of the other conditions. Hence, the remaining value reflected only the contribution of the edge-enhancement without the influence of the disruptive colouration. This calculation is made separately for the stimuli presented with and without shadows. These data are presented in Fig. 3 - plots with control times presented separately are included within the Supplementary Materials. Shapiro-Wilks tests conducted on the data from each condition did not reject the null hypothesis (alpha = 0.05) that the population was normal, except the condition with width = 8, offset = 20, on a background with shadows (W = 0.835, p = 0.038).

Search times were generally longer for the targets presented upon backgrounds without shadows – see the secondary y-axes showing seconds in Fig. 3. As the edge enhancement width and luminance offset was increased, detection times for targets on shadowed backgrounds became slower, implying improved crypsis. The effect of edge enhancement for targets on backgrounds without shadows was less consistent: for stimuli with small offsets and wider edges there was an improvement in crypsis, for all other edge manipulations there was a small decrease in crypsis.

Throughout this paper we adopt a Bayesian framework for the analysis of our data. This has the advantage of comparing the odds for both the null and experimental hypothesis, this makes the possibility of a Type-I error less likely^20^. All data and analyses are available on the open science framework^21^.

A Bayesian ANOVA (JASP^22^; see Supplementary Material) was conducted. The preferred model, with the highest Bayes factor (Log_e_(BF_10_) = 11.901), included all 2-way and 3-way interactions (between width, offset and shadow). This indicated that varying the strength (width and offset) of the edge enhancement had differing effects depending on the presence of shadows within the stimulus background. For the sake of brevity we concern ourselves here with how well each type of edge enhanced camouflage performed relative to controls placed upon the same type of background.

Bayesian t-tests (JASP^22^; see Supplementary Material) were used to determine whether edge enhancement resulted in increased reaction times relative to non-edge enhanced controls in each condition. When hidden within a shadowed background, strong to extreme evidence was found for improved camouflage in three types of edge enhanced targets over the non-edge enhanced controls: larger (40 pixel) offset and wide (16 pixel)(log_e_(BF_10_) = 6.087); small offset (20 pixel) and wide (log_e_ (BF_10_) = 3.769); Small offset and narrow (8 pixels) (log_e_ (BF_10_) = 3.305). Strong evidence was also found for improved camouflage in the target with small offset and wide edge enhancement over the non-edge enhanced targets when they were presented on a background without shadows (Log_e_(BF_10_) = 2.403). Where the mean reaction times are slightly faster than controls on the background without shadows, these differences are not supported by the (two-tailed) Bayes t-test, evidence is moderate to anecdotal for H_0_ (see Supplementary Materials). The effect size^23^ associated with these BFs is shown in the Supplementary Materials. Therefore, for the current stimuli, the cost of having enhanced edges on a background without shadows was minor when contrasted with the advantage of having such edges on shadows. These results were robust to manipulations of the Cauchy prior width (0.7–1.45; used 1.0).

## Experiment 2: Is the improved crypsis with edge enhancement due to background matching to shadows?

In Experiment 1 the edge enhanced targets were most difficult to find, relative to controls, on the shadowed background. There are two ways that edge enhancement could affect crypsis when placed upon a background containing shadows. First, the similarity between the dark edge enhancement element and the shadows may improve background matching. Second, edge enhancement may also induce a percept of pictorial relief, appearing similar to the pictorial relief created by the shadows. Experiment 2 explores these possibilities, we examine whether omitting the light or the dark component of edge enhancement would result in reduced crypsis. We also include an edge enhanced camouflage with a square profile to explore whether the shape of the gradient was important.

### Results and Discussion

As in Experiment 1, the analysis of Experiment 2 excluded errors and used log transformed and z-scored reaction times. The time taken to detect targets is shown in Fig. 4. Shapiro-Wilks tests conducted on the data from each condition did not reject the null hypothesis (alpha = 0.05) that the populations were normal.

A Bayesian ANOVA (see Supplementary Material) suggested that the preferred model, with the highest Bayes factor (Log_e_(BF_10_) = 6.870), included all 2-way and 3-way interactions (between edge-type, offset and shadow).

Bayesian t-tests were conducted to establish the effectiveness of edge enhanced camouflage (longer detection times reflect improved crypsis) relative to the control (non-edge enhanced) stimuli. As in Experiment 1, clear evidence of improved crypsis for edge enhancement stimuli relative to controls was found on the background with shadows. We found evidence of a reaction time increase when edges were ‘no-low’ with small-offset (log_e_ (BF_10_) = 1.495, *moderate evidence*) and large-offset (log_e_ (BF_10_) = 3.689, *very strong evidence*), also for the ‘no-high’ edge with large offset (log_e_ (BF_10_) = 2.875, *strong evidence*). The square edge-profile was most cryptic upon the shadowed background (log_e_ (BF_10_) = 3.812 and 3.49 respectively for small and large offset edges, *very strong evidence*). On the background without shadows, two small offset edge types had increased crypsis compared to the non-edge enhanced controls; the small offset ‘no-high’ form (log(BF_10_) = 2.589, *strong evidence*) and the small offset ‘no-low’ form (log(BF_10_) = 2.102, *moderate evidence*). The square edge-types were not cryptic relative to controls. The Bayes t-test results were robust to manipulations of the width of the Cauchy prior. See the Supplementary Materials for a full summary of the Bayesian t-test results, including effect sizes.

The results of Experiment 2 followed the same general pattern of the results of Experiment 1: edge enhancement generally improved crypsis, though the degree of improvement was dependent upon edge-offset and the presence of shadows in the background. There was no evidence of the edge enhanced stimulus ever being conspicuous relative to the control stimulus.

The ‘no-low’ edges omitted the dark band that may have provided a match to the leaf shadows, nevertheless they still offered a cryptic advantage over controls. This suggests that edge enhancement is not simply producing a background match between shadows and the dark element of edge enhancement. Rather, the edge enhancement improves crypsis, even when presented upon background without shadows. Small-offset edges were cryptic on the no-shadow background, whereas large-offset edges were not better than controls. This implies that large offset edges may increase conspicuity^24^, although there was no overall cost to this in the current study. One explanation of the results of Experiments 1 and 2 is that edge enhancement induces a percept of pictorial depth that may interfere with the integration of outline contour elements^25^. In the final study we address this issue directly by asking participants to report their perception of depth as we manipulate edge enhancement.

## Experiment 3: Does edge enhancement induce a percept of pictorial depth?

One interpretation of the previous results is that edge enhanced camouflage gives the impression of pictorial relief. If this is the case, then edge enhancement improves the match to the background by hiding the animal’s true depth and giving an impression of relief that more closely matches that of the background - i.e. matching the variable relief you might expect in leaf litter or on a pebble beach. The third experiment tested whether edge enhancement influences the perception of relative pictorial relief between a camouflage pattern and the background. We use a factorial design presenting stimuli on a background with and without shadows, while varying edge enhancement for the target camouflage. We leave a gap between the target-object and the background in order that the obvious occlusion cue does not confound the percept or relative pictorial depth, see Fig. 5 right. See methods (Experiment 3 for further details).

### Results and Discussion

Participant responses of more pictorial relief, the same relief or flatter were coded as +1, 0, −1 respectively. The means of the codified responses are shown in Fig. 5 left. The mean reaction times were ranked and then fit to a normal distribution. Shapiro-Wilks tests conducted on the data from each condition did not reject the null hypothesis (alpha = 0.05) that the population was normal.

A Bayesian ANOVA (see Supplementary Material) suggested that the preferred model, with the highest (Bayes factor (Log_e_(BF_10_) = 17.592, *extreme evidence for H*_*1*_), included only the key manipulation of edge-type. The models including shadows had a slightly lower Bayes factor (Log_e_BF_10_ = 16.421, *extreme evidence for H*_1_, for Edge Type + Shadows, and Log_e_BF_10_ = 16.076, *extreme evidence for H*_*1*_, for Edge Type + Shadows + Edge Type*Shadows.). This suggests that context (leaf shapes with or without shadows) had little influence upon depth judgements.

Bayesian t-tests found strong evidence that all of the edge enhanced targets were judged to have more pictorial relief than the non-edge enhanced control stimuli, regardless of background type (Table 1 – effect sizes are shown in the Supplementary Materials). Results were robust to manipulations of the width of the Cauchy prior.

## General Discussion

Our results suggest that edge enhancement can improve crypsis for targets (Experiment 1), and that this advantage can occur when targets are presented against backgrounds without shadows (Experiments 1 and 2) and even when the enhanced edges provide no obvious pictorial match to the background (Experiment 2). However, the profile of the edge-enhancement is important, both width and offset influence crypsis; larger offsets lead to higher contrast edges which were more cryptic on backgrounds with shadows, while lower contrast edge enhancement was more successful on backgrounds without shadows (Experiments 1 and 2). Wider edges seem to offer better crypsis (Experiment 1). Taking the results of Experiments 1 and 2 together, the most successful edge type overall was the wide, small offset no-low edge (a light edge between the dark and light blotches, see 4^th^ column Fig. 2) as this afforded a cryptic advantage on both background types.

The fact that edge enhancement improved camouflage on a shadowed background even when the edge did not contain a dark, shadow-like element suggests the mechanism is not solely due to background matching of the dark edge enhancement and shadows. Finally, edge enhancement creates a strong impression of pictorial relief (Experiment 3). We suggest that edge enhancement mimics the perceived depth patterns of natural scenes and thereby enhances camouflage. This is in line with Cott’s^3^ suggestion that pictorial relief may inhibit perception of an animal’s true form and improve background matching in settings with a variety of depth relations between objects^9^.

The pictorial relief created by edge enhancement could minimise differences in perceived pictorial relief between a camouflaged object and its background: targets without edge enhancement appear flat, allowing segregation from a background that contains pictorial relief. This suggests that in order for a target to be hidden, simple pictorial background matching may not be sufficient. Higher order properties of the scene, such as perceived depth, may also need to match. The results from Experiment 2 suggest that under some circumstances matching perceived depth might be more important than simple pixel-matching or in Thayer’s terms by utilizing “background pictures” (9, page 175). This could be viewed in different terms as matching between the perceived 3-D texture of the target and the background. In snakes, edge enhanced camouflage is associated with a passive hunting strategy, the animal hides and pounces when prey pass by unaware^26^. For this strategy to work all available visual cues to the animal’s presence need to be minimised, the animal needs to match the background in colouration, texture and in the perceived depth.

The camouflage featured in the current study is both background matching and disruptive – the texture of the target is visually similar to that of the leafy background and colours of the texture and the leaves are both sampled from the same population of colours with constraints (see Supplementary Material). We chose this common type of camouflage pattern as our control condition in a test of the effectiveness of edge enhancement. We did not test the effectiveness of edge-enhancement in a target with a single uniform colour that was broken into patches by high-contrast lines. We had supposed that such a pattern might be too conspicuous due to the largely uniform colouration. However, it is possible that edge enhancement might improve crypsis on uniform colouration, because our results show that edge enhancement does not rely solely upon improvements in the visual match between the background and the animal, dark edges can help to hide snakes even when they are hidden upon a background that does not feature shadows. We have not tested this, but it is possibly that edge enhancement may improve crypsis even without disruptive colouration.

Our study shows that edge enhancement benefits disruptive camouflage beyond background matching. The increased likelihood of perceptual segmentation that results from enhanced false edges^7^ likely plays a role, either by disrupting object recognition, or by affecting background matching in terms of edge density or spatial frequency of pattern elements. However, this does not rule out the possibility that edge enhancement also operates through additional mechanisms, both in our paradigm and in nature. The luminance gradients we used for edge enhancement in the current study are very similar to those found in the Cornsweet illusion (see ref. 27) or the watercolour illusion^28^, and they could have changed the perceived colours of the stimuli in a similar fashion. Colours bordered by dark bands would have appeared darker and colours bordered by light gradients would have appeared lighter, which could also contribute to disruptive and background matching effects.

Our study is the first to quantify experimentally the contribution of edge enhancement to disruptive camouflage. It is also the first to use a very large display screen to present participants with an ecologically valid search field. We also demonstrate that these camouflage patterns can create a perception of pictorial relief, and show the impact this has upon crypsis. We propose that edge enhancement operates through multiple mechanisms, including background matching both of the pictorial relief and in colour patterns.

## General Methods

Our study used human participants. All participants had normal vision or wore their optical prescription to correct to normal Snellen Acuity. Ethical approval for the study was granted by the Abertay University Social and Health Sciences Ethics Panel and conducted in accordance with the declaration of Helsinki (2008, Version 6). All participants gave their written informed consent to participate.

Stimuli featured a single snake-shaped camouflage-textured region set amongst a background of leaf-shaped blocks of colour (see Fig. 2). The stimuli had high edge density and colour heterogeneity to reflect natural, cluttered environments such a forest floor. We begin with a calibrated digital image of the forest floor in Kibale National Park, Uganda^29^. The image pixel colours were converted to CIE LAB format and colours beyond one standard deviation on the A and B axes were excluded. The background of each image was created by sequentially stamping a randomly orientated leaf-shaped mask 15,000 times at random locations within the image area, the colour of the leaf area was chosen randomly from the calibrated sample. The length of each leaf mask was chosen randomly from a positively skewed distribution with a minimum of 20 pixels (0.62° visual angle) and peak of 21 pixels (0.65° visual angle) 95^th^ percentile 46 pixels (1.44° visual angle). The width was chosen from a similar distribution resulting in a width that was roughly 20% of the length. Shadows were added to the background, when required, by darkening the area to the left of each leaf by 70%, this gave the impression that the scene depicted on the screen was illuminated from behind the participant’s right shoulder. The width of shadows was chosen randomly from the absolute values of a normal distribution with a mean of 2.4 and a spread of 3 pixels [matlab: abs(randn(2.4,3))]. This results in shadows with a peak width of 0.02° visual angle and falling off towards 0.23° visual angle at the 95^th^ percentile.

The disruptive colouration of the snake patterning was created by first generating a white noise image. The white-noise was then filtered in the Fourier domain (Eq. 1). Where *d* is the distance from the image centre (in Fourier-space) and μ was 0.048 and σ was 120.


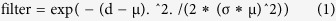


The filtered texture was then posterised by assigning one of two colour values to the values above and below the 50^th^ percentile. The colour values where chosen from the same population as that used for the leaves, with one exception. The source pixel colours were grouped according to the brightness (L in CIE-LAB space), one patch colour was chosen from those pixels with a brightness level that fell within the percentile range 35–45 and the other from the range 55 to 65. The reasoning behind this is that successful disruptive colouration requires the animal to have good contrast between its colour patches^30^ and colour match between its patterning and that of its background^2^,^5^. This gives a camouflage texture without edge enhancement (see snake in Fig. 2). A snake mask (randomly oriented, 350 pixels long, 10.95° visual angle) was then used to select an area of the snake texture. This area was copied onto the leafy background image.

### Edge enhancement

we adopt a parsimonious definition of edge enhancement, where there is a linear change in brightness from the value of the colour patch towards the most-contrasting colour at the patch edge. This is defined by two parameters; one is the *offset*, the difference in brightness from the camouflage patch towards the enhanced edge. The other parameter is *width,* the spatial-range over which the change from main patch colour to enhanced edge colour take place. So, for example, a highly contrasting enhanced edge with a fine spatial-scale might have a small width and a large offset. The snakes in Fig. 2 illustrate different forms of edge-enhancement used in Experiments 1–3. The widths used in Experiments 1 and 2 were 8 and 16 pixels respectively, corresponding to visual angles of 0.25° and 0.50° respectively.

#### Screen set-up Experiments 1 and 2

Stimuli were displayed on a 6 × 2 metre display formed-from a pair of 3 × 2 metre back projected screens that were each illuminated by a pair of Sony Brightera 3 LCD projectors. The brightness of each screen was manually linearised and matched to one another using a Minolta LS100 photometer. Once matched, a mean-grey display [RGB = 128, 128, 128] resulted in a measured colour of [25.7, 0.332, 0.381 and 27.6, 0.355, 0.374] for the left and right screen respectively [CIEcdm2, x, y] – measured with a Cambridge Research Systems ColorCal MKII colorimeter. Stimuli were presented using software written in Matlab^31^ using the Psychophysics Toolbox^32^,^33^,^34^. The room was painted matt black and only lit by the display system itself.

## Method Experiment 1

Ten participants (mean age 29.2; four female), including one of the authors (JE), were tasked with finding a snake-like target against a background of leaf-like objects. The same pattern of results and statistical significance were obtained when the author-participant was removed from the analysis. A variety of edge enhancement conditions were presented against backgrounds with and without shadows.

**Figure 1:**
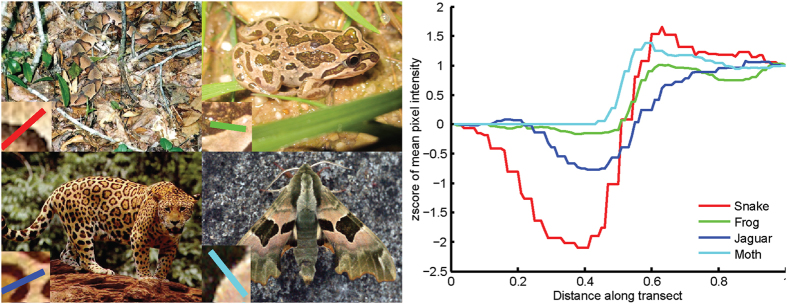
Each line drawn upon the animal photographs indicates a transect location of the data graphically presented in the right hand plot. (Right) pixel intensity values along each transect illustrated upon the photographs. (Top-Left) A copperhead snake (*Agkistrodon contortrix contortrix*), (Top-Centre) A spotted marsh frog (*Limnodynastes tasmaniensis*). (Bottom-Left) A jaguar *(Panthera onca*). (Bottom-Centre) A Lime hawk moth (*Mimas tiliae*). Images: (Snake Source wikimedia commons, Tim Ross; Jaguar, source National Digital Library, US fish and wildlife service; The spotted marsh frog, Matt Clancy; https://commons.wikimedia.org/wiki/File:Spotted_marsh_frog_(Limnodynastes_tasmaniensis)_(8236182307).jpg) is licensed under the Attribution 2.0 Generic license. Lime hawk moth (https://www.flickr.com/photos/33398884@N03/5897570808), by Ben Sale, is also licenced under the Attribution 2.0 Generic Licence. The Attribution 2.0 Generic license terms can be found on the following link: https://creativecommons.org/licenses/by/2.0/deed.en.). NB. The images shown are not calibrated to reflect the visual response of any particular organism, we have linearized the intensity values within the images by raising to the power 2.2^8^ and we then average across red, green and blue pixels within a diameter of seven pixels for each sample along the transect.

**Figure 2:**
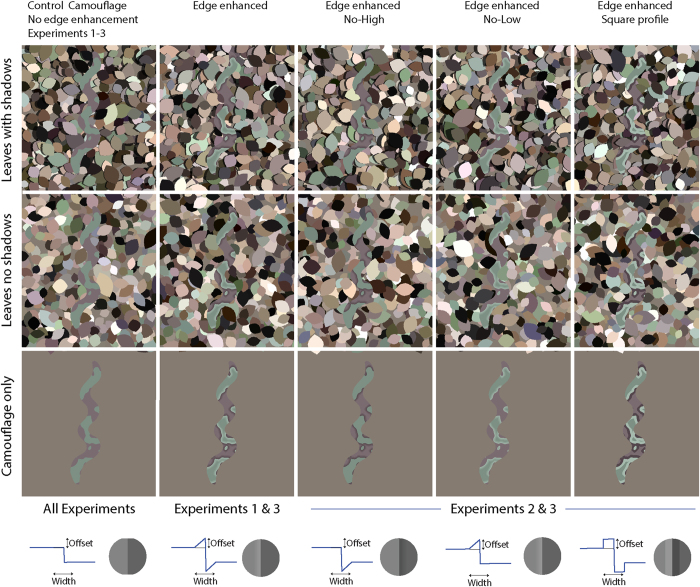
Illustration of the range of key stimulus properties. (Lower panel) camouflaged snakes featuring varying types of edge enhancement used in each study. (Upper-panel) the same snakes are placed against a background of simulated leaves casting shadows. (Middle panel) shows the snakes on the leaves, which are rendered without shadows. In individual experimental trials a single target snake was featured in each image. We adopt a parsimonious model of edge enhancement, using only two free-parameters offset and width. The offset is the change in luminance (CIE L) added to light patches or subtracted from dark patches. The width is the spatial range over which this luminance range occurs. Edge enhanced stimuli in this example have an offset of 40 and a width of 16 pixels.

**Figure 3:**
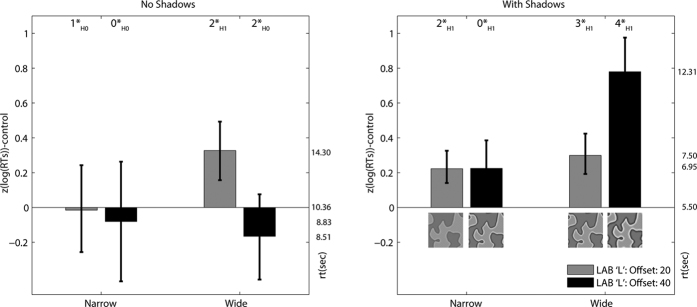
Control-relative reaction times (log transformed and z-scored) for snake detection in Experiment 1. The right-hand y-axis shows the mean reaction time for each condition (prior to the log transform and z-score). The offset is the change in luminance (CIE L) added to light patches or subtracted from dark patches. The width is the spatial range over which this luminance range occurs. All error-bars represent the bootstrapped 95% confidence intervals for the z-scored log reaction times. Inset at the top of each plot is a summary of the Bayesian t-test results, 0* = anecdotal, 1* = moderate, 2* = strong, 3* = very strong and 4* = extreme evidence for H_0_ or H_1_ (the null and experimental hypotheses respectively) – based upon Jeffreys^18^ and Lee and Wagenmakers^19^ criteria.

**Figure 4:**
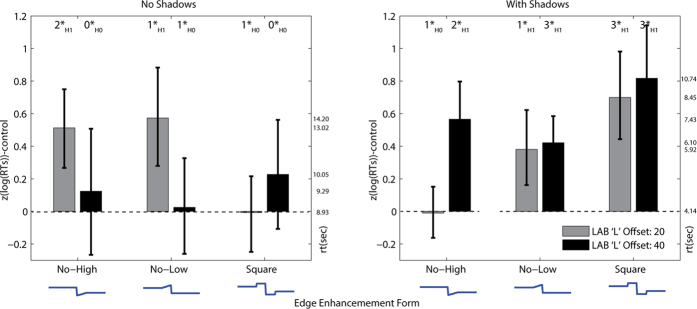
Control-relative reaction times for the detection of camouflaged targets where background shadows and types of edge enhancement are modified. Inset at the top of each plot is a summary of the Bayes t-test result (See the key in Fig. 2 caption for an explanation of the Bayes t-test significance levels). The error-bars represent the 95% bootstrapped confidence intervals for the z-scored and log reaction times.

Five levels of edge enhancement were presented; edge widths (see Fig. 1, inset) were narrow or wide (0.25° and 0.5° visual angle) combined with LAB luminance offsets of 20 or 40 or no edge enhancement. The stimuli were divided into eight equally sized rectangular regions (4 columns, 2 rows). Targets were presented with a random orientation and in a random order twice within each location (80 trials). On half of trials the background leaves featured drop shadows.

Participants could move their heads and eyes freely and indicated the location of the target using a keyboard. Participants were given 12 practice trials before starting the experiment.

## Method Experiment 2

Ten participants (mean age 28; four female) performed the same task as in Experiment 1 with variations of the edge enhancement pattern and a control non-edge enhanced target. Three differently edge enhanced targets were presented: a dark gradient without an adjacent light gradient (no-high), a light gradient without an adjacent dark gradient (no-low), and adjacent dark and light bands without a gradient (square). The edge enhancement spatial scale was fixed at 16 pixels (0.5°) and one of two luminance levels (20% and 40%). All other methodological details were the same as Experiment 1. If the ‘no-low’ stimuli prove cryptic on shadowed backgrounds then this would be strong support for the argument that these edges are not merely matching the background but rather interfering with the perceptual processes underlying object detection. The ‘square’ profile edges were included to examine whether the overall shape of the edge-profile was important.

## Method Experiment 3

**Figure 5:**
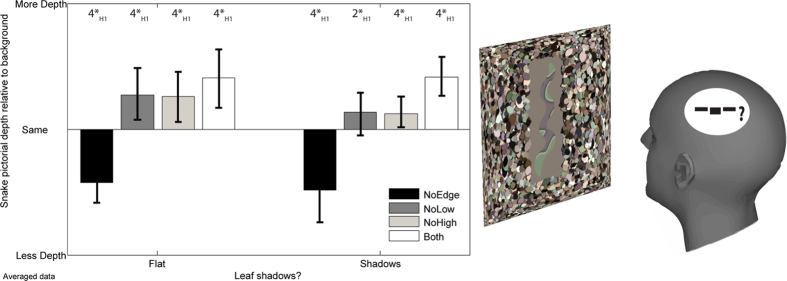
(Left) Averaged participant responses for the depth-judgement study. Participants tend to see the snake camouflage as having more pictorial relief relative to the background where edge enhancement is included within the camouflage texture. (Right) Illustration of the stimuli used.

Ten participants (mean age 31; four female), including the authors PGL and KSB, were asked to make depth judgements for three edge enhancement patterns and a control non-edge enhanced target. The same pattern of results and statistical significance were obtained when the author-participants were removed from the analysis. The snake targets were presented vertically and 13.30° long. This was surrounded by a background of 4096 overlapping leaves (26.72°). In order to remove occlusion as a depth cue the central area (4.49 × 13.80°) surrounding the target featured no leaves – see Fig. 5 (Right). Stimuli were presented upon a linearised Sony Trinitron monitor (Model GDM-F520) with a viewing distance of 64 cm. CIEcdm^2^ xy values at mid-grey (128,128,128) were [65.320, 0.284, 0.298], measured using a Cambridge Research Systems ColorCal Mk II. Stimuli were presented with software written with PsychoPy^35^.

Three types of edge enhancement were presented: both dark and light gradients (both), a dark gradient without an adjacent light gradient (no high) and a light gradient without an adjacent dark gradient (no low). The spatial scale was fixed at 0.53°. Participants were presented with 25 trials from each of the experimental conditions in a random order (200 trials).

Participants viewed the stimuli in a darkened room with their dominant eye, through an aperture that occluded the edge of the monitor. The only source of illumination within the room was the display screen itself. A chin rest was used to ensure a consistent 60 cm viewing distance. Participants were asked to judge if the snake appeared to have more pictorial depth, the same pictorial depth, or was flatter than the leafy background and responses were recorded using the keyboard. Prior to starting the experiment participants were given unlimited practice trials (minimum two per condition).

## Additional Information

**How to cite this article**: Egan, J. *et al*. Edge enhancement improves disruptive camouflage by emphasising false edges and creating pictorial relief. *Sci. Rep.* **6**, 38274; doi: 10.1038/srep38274 (2016).

**Publisher's note:** Springer Nature remains neutral with regard to jurisdictional claims in published maps and institutional affiliations.

## Supplementary Material

Supplementary Information

## Figures and Tables

**Table 1:** Bayesian t-test analysis of perceived pictorial depth relative to non-edge enhanced control stimuli.

Has shadows	Edge-type	Log_e_(BF_10_)	Error %	Support for H_1_ (Jeffreys)
0	No-low	7.460	1.257e -8	Extreme – H1
0	No-high	4.932	5.484e -8	Extreme – H1
0	Both	5.227	6.124e -7	Extreme – H1
1	No-low	5.394	5.968e -7	Extreme – H1
1	No-high	2.894	1.701e -7	Strong – H1
1	Both	5.995	5.633e -8	Extreme – H1
