# Supplementary material for: Edge enhancement improves disruptive camouflage by emphasising false edges and creating pictorial relief
Source: Sci Rep. 2016 Dec 6;6:38274. doi: 10.1038/srep38274 (PMC5138594; doi:10.1038/srep38274)
Supplement: Supplementary Information [file srep38274-s1.pdf]

Edge enhancement improves disruptive camouflage by emphasising false edges and creating pictorial relief.

Authors:

John Egan[1],

Rebecca J. Sharman[2],

Kenneth Scott-Brown[1],

Paul George Lovell[1],

[1] Abertay University, Division of Psychology, School of Social and Health Sciences, 1, Bell St, Dundee, DD1 1HG.

[2]University of Stirling, School of Psychology, Bridge of Allan, FK9 4LA.

Supplementary Material

Further Analyses

All analyses and raw data can be viewed and downloaded from the Open Science Framework see:

Lovell, P. G. (2016, September 6). Edge enhanced disruptive camouflage and its influence upon detection and depth perception. Retrieved from osf.io/me5cd

Experiment 1

Correlation between RTs and errors.

Participants responded incorrectly (selecting the wrong target location) on 8% of trials. As anticipated, participant reaction times were significantly positively correlated with error-rates. i.e. those experimental stimuli which resulted in the longest search times also resulted in the most error-trials where participants may have given-up and subsequently selected a location randomly ($r=.629$, $p = 0.051$). This suggests that participants were not trading speed for accuracy, a possible behaviour when participants are not engaged by an experiment. The redundancy between error-rates and reaction times allows us to concentrate our analyses on reaction-times only. The zScored(log reaction times) for control stimuli and each experimental condition are shown in Figure 1.

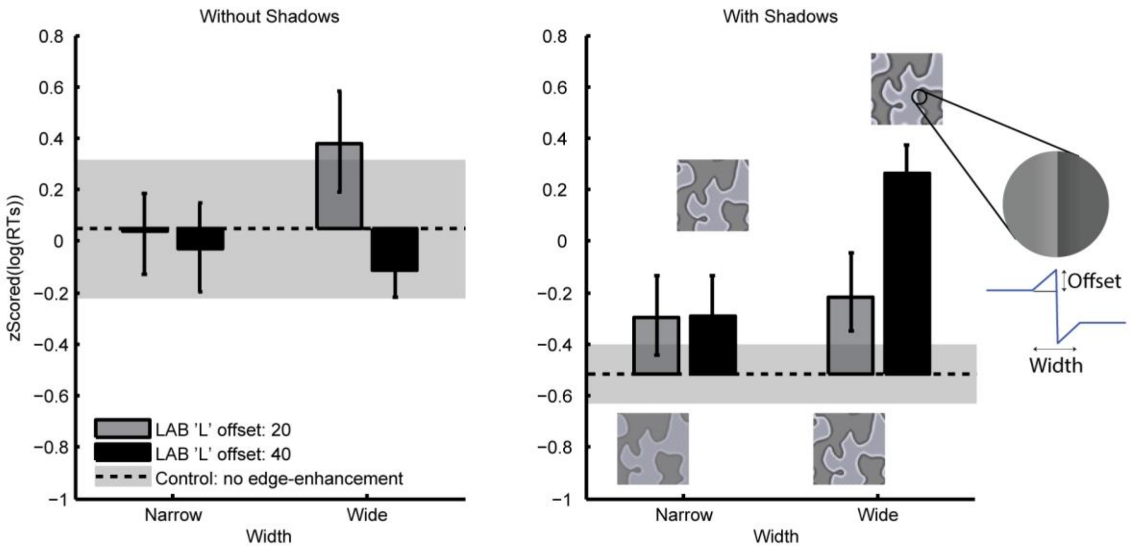

Supplementary Figure 1. Reaction times (log transformed and z-scored) for snake detection in experiment 1. The dashed horizontal lines represent the control condition means with the grey area representing the error-bars. All error-bars represent the bootstrapped 95% confidence intervals.

Bayesian ANOVA. Bayes factors are shown in the form of $Log_e(BF_{10})$. Positive values indicate that the observed results were more likely under the stated model than under the null model, while negative values indicate that the observed results are more likely under the null model than under the stated model. The models are ranked accordingly.

Table S1. Each potential Bayes ANOVA model of the reaction time data ranked in order of the Log_e(BF10).

Model	$Log_e(BF_{10})$	% Error	Support for H (Jeffreys, 1961)
Shadows + Offset + Width + Shadows*Offset + Shadows*Width + Offset*Width + Shadows*Offset*Width	11.901	2.680	Extreme – H1
Shadows + Offset + Width Shadow*Offset	10.600	2.159	Extreme – H1
Shadows + Offset + Width + Shadows*Offset + Shadows*Width	10.046	2.470	Extreme – H1
Shadows + Offset + Width + Shadows*Offset + Offset*Width	9.416	2.719	Extreme – H1
Shadows + Offset + Width + Shadow*Offset + Shadow*Width + Offset*Width	8.923	5.010	Extreme – H1
Shadows + Width	8.325	1.176	Extreme – H1
Shadows + Offset + Shadows*Offset	8.245	1.845	Extreme – H1
Shadows + Width + Shadows*Width	7.827	11.740	Extreme – H1
Shadows + Offset + Width	6.917	2.947	Extreme – H1
Shadows	6.469	0.952	Extreme – H1
Shadows + Offset + Width + Shadows*Width	6.300	3.388	Extreme – H1
Shadows + Offset + Width + Offset*Width	5.713	3.425	Extreme – H1
Shadows + Offset + Width + Shadows*Width + Offset*Width	5.085	3.142	Extreme – H1
Shadows + Offset	5.040	2.411	Extreme – H1
Width	1.200	1.260	Extreme – H1
Offset + Width	-0.253	1.822	Anecdotal – H1
Offset + Width + Offset*Width	-1.463	2.322	Anecdotal – H0
Offset	-1.470	1.085	Anecdotal – H0

Table S2. Analysis of effects for the Bayes ANOVA models presented in Table S1.

Effects	P(incl)	P(incl|data)	$BF_{Inclusion}$
width	0.737	0.983	20.530
offset	0.737	0.974	13.436
hasShadows	0.737	1.000	17366.699
width ✻ offset	0.316	0.709	5.287
width ✻ hasShadows	0.316	0.763	6.958
offset ✻ hasShadows	0.316	0.966	62.142
width ✻ offset ✻ hasShadows	0.053	0.634	31.166

Table S3. Bayesian t-test analyses of target detection times relative to non-edge enhanced control stimuli. Effect sizes calculated according to the method described by Rouder et al., (2009).

Background shadows	Width	Offset	Effect size	Log_eBF_{10}	Error %	Support for H (Jeffreys)
No	8	20	-0.30	-1.170	7.054e -5	Moderate – H0
No	8	40	-0.13	-1.095	6.131e -5	Anecdotal – H0
No	16	20	1.17	2.403	5.886e -7	Strong – H1
No	16	40	-0.40	-0.542	1.856e -5	Anecdotal – H0
Yes	8	20	1.40	3.305	1.635e -6	Strong – H1
Yes	8	40	0.81	0.925	3.114e -8	Anecdotal – H1
Yes	16	20	1.52	3.769	2.633e -7	Very strong – H1
Yes	16	40	2.23	6.087	9.561e -8	Extreme – H1

Experiment 2

Correlation between RTs and errors: The averaged overall error-rate was higher in experiment 2 (14% of trials). As with experiment 1, there was a strong positive correlation between reaction times and errors (r = 0.91, p < 0.001). Mean reaction times for each observer varied from 10 seconds to 30 seconds, with corresponding error rates of 7% and 2% respectively.

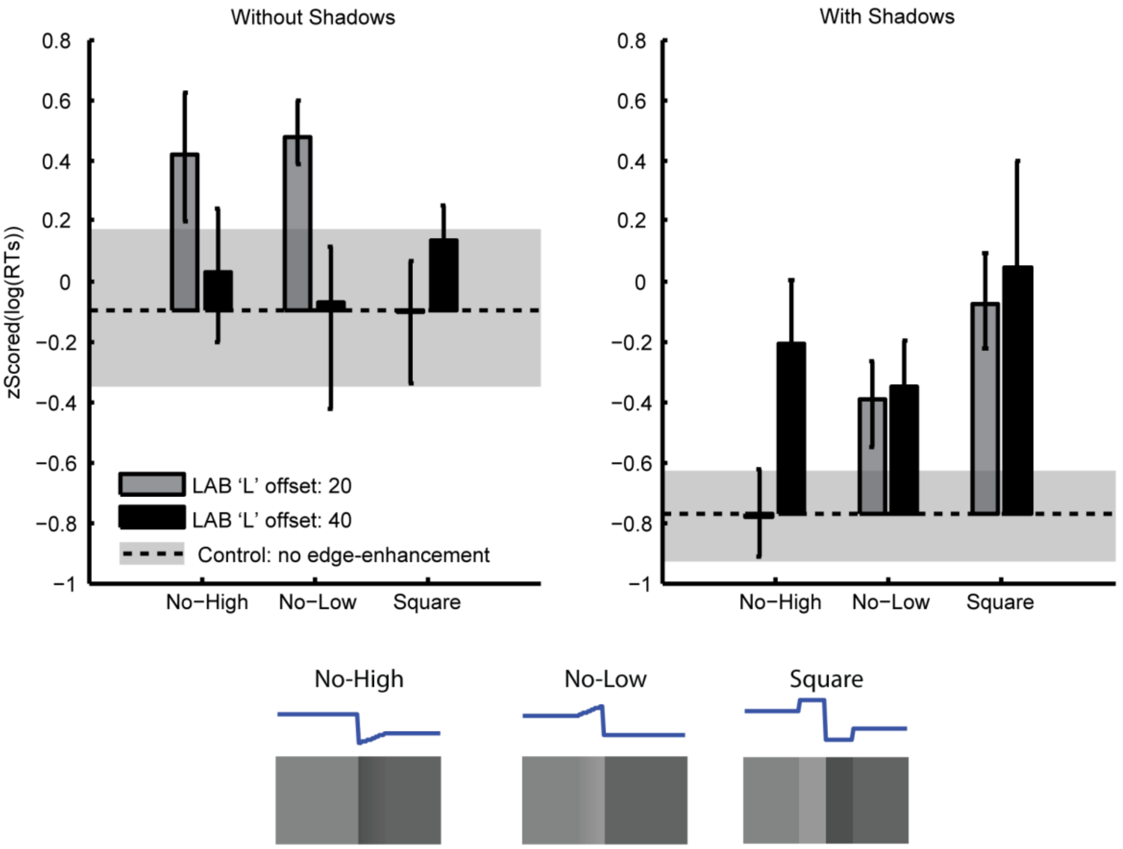

Supplementary Figure 2. Reaction times (log transformed and z-scored) for snake detection in experiment 2. The dashed horizontal lines represent the control condition means with the grey area representing the error-bars. All error-bars represent the bootstrapped 95% confidence intervals.

Table S4. Each potential Bayes ANOVA model of the reaction time data ranked in order of the Log_e(BF10).

Model	$Log_e(BF_{10})$	% Error	Support for H (Jeffreys)
Shadows + Edge Type + Offset + Shadows*Edge Type + Shadows*Offset + Edge Type*Offset + Shadows*Edge Type*Offset	6.870	5.431	Extreme – H1
Shadows + Edge Type + Offset + Shadows*Edge Type + Shadow*Offset + Edge Type*Offset	5.093	3.074	Extreme – H1
Shadows + Edge Type + Offset + Shadows*Edge Type + Shadows*Offset	4.748	2.328	Extreme – H1
Shadows + Edge Type + Shadows*Edge Type	3.505	1.408	Very strong – H1
Shadows + Offset + Shadows*Offset	3.200	41.053	Strong – H1
Shadows + Edge Type + Offset + Shadows*Edge Type + Edge Type*Offset	2.099	3.965	Moderate – H1
Shadows	1.972	1.687	Moderate – H1
Shadows + Edge Type + Offset + Shadows*Edge Type	1.900	3.692	Moderate – H1
Shadows + Edge Type + Offset + Shadows*Offset + EdgeType*Offset	0.994	2.645	Anecdotal – H1
Shadows + Edge Type + Offset + Shadows*Offset	0.903	1.939	Anecdotal – H1
Shadows + Offset	0.314	1.898	Anecdotal – H1
Shadows + Edge Type	0.135	1.126	Anecdotal – H1
Shadows + Edge Type + Offset + Edge Type*Offset	-1.433	12.501	Anecdotal – H0
Shadows + Edge Type + Offset	-1.456	3.556	Moderate – H0
Offset	-1.639	1.587	Moderate – H0
Edge Type	-1.841	0.583	Moderate – H0
Edge Type + Offset	-3.473	2.597	Very strong – H0
Edge Type + Offset + Edge Type*Offset	-3.645	2.405	Very strong – H0

Table S5. Analysis of Effects - dependent for Experiment 2.

Effects	P(incl)	P(incl|data)	Log(BF $_{Inclusion}$)
shadows	0.737	0.999	5.787
edge-type	0.737	0.981	2.922
offset	0.737	0.966	2.311
shadows ✻ edge-type	0.316	0.976	4.466
shadows ✻ offset	0.316	0.953	3.786
edge-type ✻ offset	0.316	0.857	2.560
shadows ✻ edge-type ✻ offset	0.053	0.710	3.788

Table S6. Bayesian t-test results comparing the non-edge enhanced control stimuli with the various edge enhanced counterparts. Effect sizes calculated according to the method described by Rouder et al., (2009).

Background shadows	Edge-Type	Offset	Effect size	$Log_e(BF_{10})$	Error %	Support for H (Jeffreys)
No	No-high	20	1.22	2.589	2.474e -7	Strong – H1
No	No-high	40	0.19	-1.019	5.406e -5	Anecdotal – H0
No	No-low	20	1.10	2.201	1.014e -6	Moderate – H1
No	No-low	40	0.05	-1.164	6.972e -5	Moderate – H0
No	Square	20	-0.02	-1.174	6.796e -5	Moderate – H0
No	Square	40	0.40	-0.553	1.893e -5	Anecdotal – H0
Yes	No-high	20	-0.04	-1.169	7.042e -5	Moderate – H0
Yes	No-high	40	1.29	2.875	2.431e -7	Strong – H1
Yes	No-low	20	0.95	1.495	4.021e -6	Moderate – H1
Yes	No-low	40	1.51	3.689	6.209e -7	Very Strong – H1
Yes	Square	20	1.54	3.812	1.083e -7	Very Strong – H1
Yes	Square	40	1.45	3.498	1.436e -6	Very Strong – H1

Experiment 3

A Bayes ANOVA was conducted to estimate the evidence-for the effect of each experimental manipulation (edgeType, presence of shadows in background) upon participant judgements of depth. The manipulation of edge-type had the strongest evidence in support of this influencing participant depth-judgements ($\log_e(BF_{10})$= 17.586). There was moderate evidence *against* the inclusion of shadows alone ($\log_e(BF_{10})$= -1.308). Even when included with shadows (as either an interaction or otherwise) evidence for inclusion was weaker than for edgeType alone.

Table S7. Each potential Bayes ANOVA model of the depth response data for Experiment 3 ranked in order of the Log_e(BF10).

Model Comparison - dependent

Models	P(M)	P(M|data)	$Log_e(BF_M)$	$Log_e(BF_{10})$	% error	Support for H (Jefferys)
Null model (incl. subject)	0.200	1.497e -8	-16.631	0.000		
EdgeType	0.200	0.654	2.022	17.592	0.703	Extreme H1
shadows	0.200	4.074e -9	-17.932	-1.301	1.687	Moderate H0
EdgeType + shadows	0.200	0.203	0.017	16.421	1.219	Extreme H1
EdgeType + shadows + EdgeType ✻ shadows	0.200	0.144	-0.400	16.076	1.407	Extreme H1

Note. All models include subject.

Table S8. Analysis of Effects for Experiment 3.

Effects	P(incl)	P(incl|data)	$Log_e(BF_{Inclusion})$	$Log_e(BF_{Backward})$	% errorB	$Log_e(BF_{Forward})$	% errorF
EdgeType	0.600	1.000	17.371	-1.171	1.407	17.592	1.687
shadows	0.600	0.314	-1.041	-17.723	2.081	-1.301	0.703
EdgeType ✻ shadows	0.200	0.042	-0.400	17.723	2.081	-0.345	1.862

Finally each edge-type is examined using a Bayes t-test to test whether depth assessments are greater than zero (where zero is equivalent to participants stating that "the depth of the snake pattern is the same as that of the background"). Here all conditions have strong to extreme evidence for there being a difference (see table S9 below).

Table S9. Bayesian t-test analysis of perceived pictorial depth relative to non-edge enhanced control stimuli. Effect sizes calculated according to the method described by Rouder et al., (2009).

Has shadows	Edge-type	Effect Size	$Log_e(BF_{10})$	Error %	Support for H (Jeffreys)
0	No-low	2.72	7.460	1.257e -8	Extreme – H1
0	No-high	1.86	4.932	5.484e -8	Extreme – H1
0	Both	1.95	5.227	6.124e -7	Extreme – H1
1	No-low	2.00	5.394	5.968e -7	Extreme – H1
1	No-high	1.28	2.849	1.701e -7	Strong – H1
1	Both	1.97	5.995	5.633e -8	Extreme – H1
